# *Haemophilus influenzae* type b vaccine formulation and risk of childhood leukaemia

**DOI:** 10.1038/sj.bjc.6600489

**Published:** 2002-08-27

**Authors:** F Groves, D Sinha, A Auvinen

**Affiliations:** Department of Biometry and Epidemiology, Medical University of South Carolina, Charleston, South Carolina, SC 29425-0835, USA; Finnish Cancer Registry, Liisankatu 21 B, FIN-00170 Helsinki, Finland; School of Public Health, University of Tampere, FIN-33014 Tampere, Finland

**Keywords:** leukaemia, child, vaccinations, *Haemophilus influenzae* type b, clinical trials

## Abstract

Incidence of childhood leukaemia was studied among subjects of a vaccine trial in Finland comparing the polysaccharide–diptheria toxoid conjugate and oligosaccharide–CRM197 conjugate *Haemophilus influenzae* type b conjugate vaccine formulations. Eighty cases of childhood leukaemia were detected: 35 among children on the polysaccharide–diptheria toxoid conjugate arm, and 45 among children on the oligosaccharide–CRM197 conjugate arm, which was not statistically significant.

*British Journal of Cancer* (2002) **87**, 511–512. doi:10.1038/sj.bjc.6600489 www.bjcancer.com

© 2002 Cancer Research UK

## 

A large case–control study conducted in the United States raised the possibility that conjugate vaccine against *Haemophilus influenzae* type b (Hib) was inversely associated with the risk of childhood leukaemia (Groves *et al*, 1999). Re-analysis of data from an earlier clinical trial in Finland (Eskola *et al*, 1990) suggested a non-significant protective effect of early versus late administration of a Hib conjugate vaccine (Auvinen *et al*, 2000). The present study aimed to evaluate the effect of different formulations of a conjugate Hib vaccine by exploiting a rare opportunity to compare different formulations within the setting of an intervention trial was provided by a nation-wide vaccination trial conducted in Finland in the 1980s (Peltola *et al*, 1994). This approach minimises the effects of bias and confounding due to known and unknown risk factors.

## MATERIALS AND METHODS

A nation-wide vaccine trial compared vaccination against *Haemophilus influenzae* type b (Hib) with a polysaccharide–diphtheria toxoid conjugate (PRP-D) versus an oligosaccharide–CRM197 conjugate (HbOC) (Peltola *et al*, 1994). The PRP-D vaccine consisted of heat-sized Hib capsular polysaccharide coupled to diphtheria toxoid (Schneerson *et al*, 1980). All 125 129 children born in Finland between 1 September 1987 and 31 August 1989 were enrolled, with a participation rate of 93.8%. The children were allocated to the trial arms based on the date of birth. All children with an odd day of birth were assigned to the PRP-D arm, while children with an even day of birth were assigned to the HbOC arm; all children received three doses of Hib vaccine: the first at age 4 months, the second at age 6 months, and the third (booster) dose between 14 and 18 months of age. All the children also received standard vaccination regime including tuberculosis (BCG), diphtheria–pertussis–tetanus (DPT), inactivated polio (IPV) and measles–mumps–rubella (MMR) vaccine.

Information on the number of boys and girls born on odd and even dates during the periods of interest were obtained from the Finnish Population Registry. Also, the number of children alive in each group by year and sex was acquired. The length of follow-up (through 1999) was 10–12 years among children in the trial (born 1987–1989). All cases of childhood leukaemia diagnosed in Finland were identified from the Finnish Cancer Registry, which is a nation-wide, population-based cancer registry (Teppo *et al*, 1994). Complete coverage of the cancer registry was ensured by crosschecking the case lists at all hospitals treating childhood leukemia and from mortality records. All childhood leukaemia cases were histologically confirmed with bone marrow aspirate.

Poisson regression analysis of leukaemia incidence rates was conducted using the number of cases as the outcome variable, with age, sex and cohort year of birth as covariates (Breslow and Day, 1987). For comparison of the PRP-D arm and the HbOC arm, a binary variable based on odd or even date of birth was used. Because of the shape of the age-incidence curve for childhood leukaemia, rising to a peak in the early preschool years, then falling before rising again, the square and cube of the age terms were also entered into the model. Separate models were fitted for acute lymphoblastic leukaemia, all other leukemias combined, and all leukaemias combined. The sex and cohort terms were not statistically significant, and were removed from all three models. The three age terms were statistically significant for the models of acute lymphoblastic leukaemia and total leukaemia, but were removed from the model for other leukaemias because they were not statistically significant.

## RESULTS

A total of 80 leukaemia cases were diagnosed from birth through age 12 among subjects born during the trial period (1987–1989). Of them, 35 were observed among children born on an odd date, i.e., belonging to the PRP-D arm, while 45 were diagnosed among children on the HbOC arm. This corresponds to a relative risk of 1.14 (95% CI 0.63–2.08) for subjects in the HbOC group. Sixty-nine of the cases were acute lymphoblastic leukaemias (ALL). Among these cases, 30 were in the PRP-D and 39 in the HbOC arm of the trial. The corresponding relative risk for the HbOC arm was 1.01 (95% CI 0.53–1.93).

## DISCUSSION

There was no suggestion of different risks of childhood leukaemia among Finnish children who received the PRP-D or HbOC formulations of *Haemophilus influenzae* type b conjugate vaccine. The design of the trial with allocation of intervention based on date of birth and prospective follow-up minimised the potential for bias and confounding (Eskola *et al*, 1990; Peltola *et al*, 1994). Very high participation rate reduced exposure misclassification to a minimum and complete ascertainment of cases was achieved by using the data from the Finnish Cancer Registry (Teppo *et al*, 1994), based on clinical, pathological and cause of death records. However, we were not able to verify if the vaccination indeed took place for a given individual, even though it was very likely given the participation rate of 93.8%. Lack of an effect in this analysis could reflect similar effect of conjugate vaccines administered at an early age. Alternatively, our findings can be taken to suggest that the earlier results did not represent a causal relationship, i.e. lack of protective effect of *Haemophilus influenzae* conjugate vaccination against childhood leukaemia.
